# ENVirT: inference of ecological characteristics of viruses from metagenomic data

**DOI:** 10.1186/s12859-018-2398-5

**Published:** 2019-02-04

**Authors:** Duleepa Jayasundara, Damayanthi Herath, Damith Senanayake, Isaam Saeed, Cheng-Yu Yang, Yuan Sun, Bill C. Chang, Sen-Lin Tang, Saman K. Halgamuge

**Affiliations:** 1 School of Public Health and Community Medicine, University of New South Wales, Randwick, NSW 2052 Australia; 2 Optimisation and Pattern Recognition Research Group, Department of Mechanical Engineering, Melbourne School of Engineering, The University of Melbourne, Parkville, VIC 3010 Australia; 3 Department of Computer Engineering, University of Peradeniya, Peradeniya, Sri Lanka; 4 Biodiversity Research Center, Academia Sinica, Nan-Kang, Taipei 11529 Taiwan; 5 Yourgene Bioscience, No. 376-5, Fuxing Rd., Shu-Lin District, New Taipei City, Taiwan; 6 Research School of Engineering, College of Engineering and Computer Science, The Australian National University, Canberra, ACT 2601 Australia

**Keywords:** Richness estimation, Viral metagenomics, Average genome length

## Abstract

**Background:**

Estimating the parameters that describe the ecology of viruses,particularly those that are novel, can be made possible using metagenomic approaches. However, the best-performing existing methods require databases to first estimate an average genome length of a viral community before being able to estimate other parameters, such as viral richness. Although this approach has been widely used, it can adversely skew results since the majority of viruses are yet to be catalogued in databases.

**Results:**

In this paper, we present ENVirT, a method for estimating the richness of novel viral mixtures, and for the first time we also show that it is possible to simultaneously estimate the average genome length without a priori information. This is shown to be a significant improvement over database-dependent methods, since we can now robustly analyze samples that may include novel viral types under-represented in current databases. We demonstrate that the viral richness estimates produced by ENVirT are several orders of magnitude higher in accuracy than the estimates produced by existing methods named PHACCS and CatchAll when benchmarked against simulated data. We repeated the analysis of 20 metavirome samples using ENVirT, which produced results in close agreement with complementary *in virto* analyses.

**Conclusions:**

These insights were previously not captured by existing computational methods. As such, ENVirT is shown to be an essential tool for enhancing our understanding of novel viral populations.

**Electronic supplementary material:**

The online version of this article (10.1186/s12859-018-2398-5) contains supplementary material, which is available to authorized users.

## Background

Viruses account for the significant majority of Earth’s biota and are vital in shaping our biosphere, but just as critically are causative agents of a plethora of plant, animal and human diseases. Despite their abundance, we are still only beginning to understand their overarching ecological roles, with the vast majority of viruses yet to be discovered. Due to the absence of conserved marker genes such as the 16S rRNA gene found in bacteria, which has been used to identify bacterial species as well as their phylogeny [1], early in vitro approaches have been limited to analyzing individual viruses in isolation [2, 3]. However, viral populations often co-occur depending on their host or environment and holistic approaches are required to understand their overall functionality. Recent attempts to study such viral mixtures using metagenomics have provided significant insights into the dynamics between viral communities, their hosts and their environment. With the rapid development of metagenomics protocols tailored toward viral mixtures, modern computational approaches can now infer various ecological parameters, such as: species abundance, richness, the Shannon-Weiner Index [4], and population evenness [5–7], directly from next-generation sequencing data (i.e. the “metavirome”). These inferred parameters provide essential information that can be used to probe deeper into the population dynamics of viral communities. Developing computational models that can produce robust and unbiased estimates of these parameters, however, is non-trivial.

For instance, PHACCS [8] uses an extended Lander-Waterman model [9, 10] to predict theoretical distributions of virome data that are compared to a distribution of observed virome data. The ecological parameters of the underlying viral populations are thus inferred when the difference between a theoretical distribution generated by PHACCS and the observed distribution is minimal. Other methods, such as CatchAll, take a similar approach but use different representations of virome data and operate under a different set of assumptions about the relationship between the underlying viral populations and the observed metagenomic data [11]. Other methods include those which rely on information in existing genomic databases, and are best applied when samples are known to contain viral types that are represented in these databases ([12–14]). In general, PHACCS and its extensions thereof, are the most widely used and are the best performing [15, 16]. However, the limitation of these methods is the assumption that the average genome length of a viral mixture in an uncharacterized sample is known, which in reality is not the case. This can lead to potentially erroneous or misleading results if an incorrect average genome length of a virome is assumed [8]. Consequently, these methods are paired with complementary methods to infer an average genome length of a virome [5, 6, 13, 17].

These complementary methods infer an average genome length using three broad approaches. The first approach makes an assumption that similar viral genomes are in similar environments, and uses the average genome length of known viruses in those environments as input to PHACCS (i.e. 50 kbp for marine viruses) [10, 16, 18]. This approach does not hold for the vast majority of viromes, since the variation in genome length can be quite large between viruses of similar environments (predominantly distributed from 1.2 kbp - 2.5 Mbp, based on 4991 viral genomes catalogued by NCBI). The second approach uses database-driven computational methods such as GAAS ([5, 6, 13, 17]) to infer an average genome length based on sequence similarity to existing viral genomes. These methods are heavily biased due to the under-representation of novel viral types in current databases. The third approach is to use in vitro methods, including: Transmission Electron Microscopy (TEM), traditional culture-based approaches, or techniques such as Pulsed Field Gel Electrophoresis (PFGE) [18–20]. PFGE is considered to be the gold standard in experimentally determining the length of DNA molecules, but requires a relatively large volume of DNA [19, 21] and is biased toward the more abundant viruses in a sample (i.e. dependent on the relative concentration of DNA per viral type). As such, these methods are not ideal for estimating the average genome length of a virome, leaving PHAACS and its derivatives poorly equipped to analyze the virome of environmental samples.

In this paper, we present ENVirT, a database-independent algorithm which estimates ecological parameters, including the viral richness, and for the first time also provides a simultaneous estimate of the average genome length. The formulation of ENVirT extends the original PHACCS model, and introduces a novel 4-dimensional heuristic optimization algorithm based on the Genetic Algorithm in combination with a unique niching strategy to arrive at estimates of both viral richness and average genome length. ENVirT requires only virome data as input, and does not rely on any other information or external databases during parameter estimation, which makes it better suited to analyzing experimental samples that typically contain novel viruses.

We also show that re-analysis of 20 virome samples from a diverse set of environments and sampling experiments produces novel insights into the respective viral mixtures that were previously not captured when analyzed using PHACCS.

## Methods

ENVirT is based on a novel 4-dimensional heuristic optimization algorithm to simultaneously estimate viral richness, evenness and for the first time the average genome length of a virome. It is formulated as an extension to the original PHACCS algorithm. The proposed extensions allow ENVirT to perform faster and independently of other databases required by PHACCS. The subsequent derivation of the ENVirT algorithm uses the following notation:

*M* denotes the number of genotypes (richness); *L* denotes the average genome length of each genotype (bp); *f*_*i*_ represents the relative abundance of the *i*^*th*^ genotype (*i*∈1,…,*M*), where *i* is the abundance rank of a genotype after they have been sorted based on their relative abundance; *R* denotes the total number of reads in a metavirome, and *r* is the corresponding average read length (bp); *o* denotes the minimum overlap for assembling reads (bp); (*C*_1_,*C*_2_,*C*_3_,…,*C*_*R*_) denotes the observed contig spectrum, where *C*_*q*_ (*q*∈1,2,3,…,*R*) is the observed number of contigs that comprise *q* reads (e.g. *C*_1_ is the number of singletons, *C*_2_ is the the number of contigs each having 2 reads, etc.); and *O*_*q*_=*q*.*C*_*q*_ is the number of reads that form contigs that comprise *q* reads (*q*∈1,2,3,…,*R*). An important assumption made in this formulation is that the *f*_*i*_s follow one of the four theoretical distributions: power-law, exponential, logarithmic or lognormal, as defined in Eqs. 1, 2, 3 and 4 respectively where *d* denotes the distribution specific real valued parameter. This assumption is justified in [22–24].

1 $$ f_{i} = \frac{i^{-d}}{{\sum\nolimits}_{j=1}^{M} j^{-d}}  $$

2 $$ f_{i} = \frac{exp(-i.d)}{{\sum\nolimits}_{j=1}^{M} exp(-j.d)}  $$

3 $$ f_{i} = \frac{(log(i+1))^{-d}}{{\sum\nolimits}_{j=1}^{M} (log(j+1))^{-d}}  $$

4 $$ f_{i} = \frac{exp(m_{i}.d)}{{\sum\nolimits}_{j=1}^{M} exp(m_{j}.d)}  $$

$m_{i} = \frac {M}{\sqrt {2\pi {}}}.\left (exp\left (\frac {-t_{i}^{2}}{2}\right)-exp\left (\frac {-t_{i+1}^{2}}{2}\right)\right)$

*t*_1_=−*∞*, *t*_*M*+1_=+*∞*,

$t_{i+1} = \sqrt {2}.erf^{-1}\left (\frac {2}{M} + erf\left (\frac {t_{i}}{\sqrt {2}}\right)\right)$ where *d*≥0 and *j*∈{1,2,…,*M*}, *erf* denotes the error function and *erf*^−1^ denotes the inverse error function.

All four functional forms of *f*_*i*_ (i.e. Eqs. 1, 2, 3 and 4) depend on *M* and a distribution specific parameter *d*. Let us denote the function defining the relative abundance of the *i*^*th*^ genotype as *F*_*i*_(*M*,*T*,*d*) where *T* denotes the distribution function given by Eqs. 1, 2, 3 or 4. Once *M*, *T* and *d* are known, the relative abundance of each genotype contained in the virome can be calculated.

Following the derivation in [10], if the expected number of reads contributing to contigs having exactly *q* number of reads is *E*_*q*_ (*q*∈{1,2,3,…,*R*}):

5 $$ E_{q} = \sum\limits_{i=1}^{M} F_{i}(M,T,d).R.q.p_{i}^{(q-1)}.(1-p_{i})^{2}  $$

where,

6 $$ p_{i} = 1-exp\left(-(r-o).F_{i}(M,T,d).\frac{R}{L}\right)  $$

Accordingly, the expected contig spectrum of a metagenome having population parameters *M*,*L*,*T*,*d* and, sequenced and assembled with parameters *R*,*r*,*o* is:

$\left (\frac {E_{1}}{1},\frac {E_{2}}{2},\frac {E_{3}}{3},\ldots,\frac {E_{R}}{R}\right)$. Given the values of *R*,*r*,*o* and (*O*_1_,*O*_2_,*O*_3_,…,*O*_*R*_), our aim is to find *M*,*L*,*T* and *d* such that the difference between (*O*_1_,*O*_2_,*O*_3_,…,*O*_*R*_) and (*E*_1_,*E*_2_,*E*_3_,…,*E*_*R*_) is minimum. Similar to [8, 10] we use the variance weighted squared difference between (*O*_1_,*O*_2_,*O*_3_,…,*O*_*R*_) and (*E*_1_,*E*_2_,*E*_3_,…,*E*_*R*_) denoted by *S*(*M*,*L*,*T*,*d*) as the similarity measure between the observed and expected contig spectra.

The ENVirT formulation is thus the minimization of the error between an expected contig spectrum *E* and the experimentally observed contig spectrum *O*, which is represented by an error function *S*:

7 $$ S(M,L,T,d) = \sum\limits_{q=1}^{R} \frac{(O_{q}-E_{q})^{2}}{V_{q}^{2}}  $$

where,

8 $$ V_{q}^{2} \,=\,\!\! \sum\limits_{i=1}^{M}\!F_{i}(M,T,d).R.q.p_{i}^{(q-1)\!}.(1-p_{i})^{2}.\!\left(\!\!1\!\,-\,q.p_{i}^{(q-1)}.(1\!\,-\,\!p_{i})^{2}\!\right)  $$

This error function *S* has multiple local minima but one global minimum (see Additional file 1). PHACCS now assumes that *L* is known, thereby greatly simplifying the optimization problem.

However, there are undesirable consequences to this assumption as an incorrect value of *L* has been reported to cause wild fluctuations in the estimation of any ecological parameter [8]. We propose that since *L* is unknown for any given real-world data set, *L* should be treated as such and instead estimated during the minimization of *S*.

The landscape of *S* is such that an optimal solution can be found using brute force but is subject to multiple local minima. We propose an optimization scheme based on the standard Genetic Algorithm (GA), which uses a heuristic approach to explore the parameter landscape of *S*. GA has been widely used in the scientific community to solve combinatorial optimization problems and since *M* and *L* are integers and *S* is non-linear in our problem formulation, GA is well suited to minimize *S*. However, since GA is also susceptible to local optima, we adopt a niching strategy [25] as follows: we first applied niching along the dimension of *T* for each of the four candidate distributions (see Additional file 1); we then applied niching along the dimension of *L* for each subspace of *T* separating the search space further into *N*_*L*_ sub-spaces. Table 1 shows that when ENVirT is applied with this niching strategy the algorithm is better able to find an optimal solution.

**Table 1:** Performance of ENVirT in comparison to standard GA algorithm on simulated contig spectra

Input parameters (expected result)						Estimated values by ENVirT					Estimated values by GA without niching				
*L*_0_	*M*_0_	*T*_0_	*d*_0_	Evenness	*f*_*max*_	*L*	*M*	*T*	*d*	*S*_*min*_	*L*	*M*	*T*	*d*	*S*_*min*_
12500	300	exp	0.030	0.790	2.956%	12500	300	exp	0.030	0.00x10 ^0^	39500	12400	exp	0.095	3.49x10 ^-2^
12500	1000	log	0.900	0.995	0.661%	14972	838	log	0.893	6.56x10 ^-3^	310000	100	lgn	1.063	2.59x10 ^1^
12500	5000	lgn	2.500	0.655	11.849%	12500	5000	lgn	2.500	0.00x10 ^0^	12500	5000	lgn	2.500	0.00x10 ^0^
12500	10000	pl	0.700	0.913	1.997%	12500	10000	pl	0.700	0.00x10 ^0^	29500	1400	log	1.911	6.38x10 ^0^
50000	300	exp	0.030	0.790	2.956%	50000	300	exp	0.030	0.00x10 ^0^	41000	100	pl	0.378	1.53x10 ^1^
50000	1000	log	0.900	0.995	0.661%	50000	1000	log	0.900	0.00x10 ^0^	100500	600	lgn	0.531	3.48x10 ^-2^
50000	5000	lgn	2.500	0.655	11.849%	50000	5000	lgn	2.500	0.00x10 ^0^	50000	5100	lgn	2.506	1.92x10 ^-2^
50000	10000	pl	0.700	0.913	1.997%	52787	10175	pl	0.707	1.72x10 ^-3^	41000	9800	pl	0.677	2.22x10 ^-2^
125000	300	exp	0.030	0.790	2.956%	125000	300	exp	0.030	0.00x10 ^0^	58500	11000	exp	0.014	2.70x10 ^-2^
125000	1000	log	0.900	0.995	0.661%	125000	1000	log	0.900	0.00x10 ^0^	69000	1800	log	0.943	3.94x10 ^-4^
125000	5000	lgn	2.500	0.655	11.849%	125000	5000	lgn	2.500	0.00x10 ^0^	125000	5000	lgn	2.500	0.00x10 ^0^
125000	10000	pl	0.700	0.913	1.997%	116341	9824	pl	0.691	1.96x10 ^-4^	203000	15000	lgn	1.922	9.34x10 ^-1^
300000	300	exp	0.030	0.790	2.956%	300000	300	exp	0.030	0.00x10 ^0^	67000	400	lgn	0.543	5.36x10 ^-2^
300000	1000	log	0.900	0.995	0.661%	217303	1373	log	0.899	1.26x10 ^-7^	156000	1900	log	0.931	1.93x10 ^-5^
300000	5000	lgn	2.500	0.655	11.849%	300000	5000	lgn	2.500	0.00x10 ^0^	310000	7400	lgn	2.635	1.09x10 ^-1^
300000	10000	pl	0.700	0.913	1.997%	277000	9800	pl	0.690	3.00x10 ^-5^	77000	5600	log	1.658	2.97x10 ^-2^

Contig spectra were generated with parameters: *R*=10000, *r*=100*bp* and *o*=35*bp*. pl = power-law distribution, exp = exponential distribution, log = logarithmic distribution and lgn = lognormal distribution. *f*_*max*_= relative abundance of the dominant genotype. *S*_*min*_= the value of the cost function corresponding to the estimated values of *M*,*L*,*T* and *d*. GA = Genetic Algorithm. We chose *M*_*LB*_=1,*M*_*UB*_=15000,*L*_*LB*_=10000,*L*_*UB*_=310000,*d*_*LB*_=0.01 and *d*_*UB*_=5 for both ENVirT and GA without niching. In order to apply the second niching strategy of ENVirT, we chose *N*_*L*_=29

### Optimization procedure

Inputs to ENVirT are the observed contig spectrum (*C*), number of reads in the virome (*R*), average read length in base-pairs (*r*), minimum overlap considered in assembling reads (*o*) and the boundaries of the domain within which values for *M*,*L*,*T* and *d* should be searched for. These boundaries are denoted the subscripts _*LB*_ and _*UB*_, which correspond to the lower and upper bound of a variable, respectively. ENVirT outputs estimates for *M*,*L*,*T* and *d*, along with a residual model error denoted by *S*_*min*_ (i.e. the minimum value of Eq. 7). These estimates are obtained by iteratively performing the following steps:

1. For each of the candidate distributions along the niched dimension *T*, perform steps S1-S3:
2. Choose a value for *N*_*L*_ to apply the second niching strategy. In this step, the *L* axis is divided into *N*_*L*_ number of overlapping windows having a constant window width. The window width (*W*_*L*_) along the *L* dimension is calculated as follows.
  $W_{L} = \frac {2(L_{UB}-L_{LB})}{\left (N_{L}+1\right)}$.
  Let *W*_*sp*_(*j*) and *W*_*ep*_(*j*) be the starting and ending positions respectively of the *j*^*th*^ window (*j*∈1,…,*N*_*L*_) along the *L* dimension. Then,
  *W*_*sp*_(1)=*L*_*UB*_
  $W_{sp}(j+1) = W_{sp}(j) + \frac {1}{2}.W_{L}$ for *j*=1,…,(*N*_*L*_−1)
  *W*_*ep*_(*j*)=*W*_*sp*_(*j*)+*W*_*L*_ for *j*=1,…,*N*_*L*_
  This definition ensures that an overlap of $\frac {1}{2}.W_{L}$ exists between each consecutive pair of windows such that the *L* values occurring along a boundary in one window occur in the middle of the next window. This property is important to avoid the possible negligence of boundary values by GA.
3. Perform GA to find the minimum of *S*(*M*,*L*,*T*,*d*) (i.e. the cost function for GA) within each of the *N*_*L*_ number of sub-spaces where the *j*^*th*^ subspace is defined by:
  *M*_*LB*_≤*M*≤*M*_*UB*_
  *W*_*sp*_(*j*)≤*L*≤*W*_*ep*_(*j*) where (*j*∈1,…,*N*_*L*_)
  *d*_*LB*_≤*d*≤*d*_*UB*_
4. Out of the 4*N*_*L*_ solutions obtained by performing GA on 4*N*_*L*_ number of sub-spaces, identify *M*,*L*,*T* and *d* corresponding to the solution with the minimum cost function (i.e. *S*(*M*,*L*,*T*,*d*)) value.

### Practical considerations

For the practical application of the ENVirT algorithm, we treat *d* as a discrete variable with a step size of 0.01. Moreover, we do not observe contig spectra with nonzero values for *C*_*R*_. In fact, the maximum *q* with a non-zero value for *C*_*q*_ is much less than *R* in real-world metagenomes. Therefore, depending on the length of the observed contig spectrum, we can safely consider a value much less than *R* for *q*_*max*_ in the actual calculation, and apply a cutoff to the maximum length of the spectrum. We recommend discretizing *M* with a minimum step size of $\phantom {\dot {i}\!}10^{(ceiling(log(M_{UB}))-2)}$ when *log*(*M*_*UB*_)>2. We also recommend discretizing *L* with a minimum step size of 0.025 *W*_*L*_. Once a solution is found in the discretized search space, we iteratively reduce the step size and repeat the optimization procedure. In its current formulation, ENVirT produces relatively accurate parameter estimates when the variation of genome lengths *L* satisfies −*log*(*v*)>2 in simulated data. Filtering non-viral DNA in vitro prior to sequencing using DNase to remove free DNAs prior to viral DNA isolation, or using a computational method post-sequencing, could improve the integrity of downstream processing by ENVirT. An example of the latter is to map the sequences against an existing database such as GenBank to identify non-viral DNA sequences [26]. However, it could be challenging for environments lacking host genomic information.

For the convenience of users, the software bundle containing the algorithm implementation is available for download at https://github.com/senanayaked/ENVirT.git. The scripts are available in Matlab (MathWorks,Massachusetts, USA) which can be executed via a user-friendly graphical user interface. Data and instructions for a sample execution of the algorithm are contained in the README.md file available with this download.

### Experimental metavirome data

Table 2 summarizes the 20 publicly available experimental virome that were analyzed in this study. The objective of selecting these datasets for analysis was to capture a variety of sampling environments, protocols and viral populations with which to validate the utility of ENVirT.

**Table 2:** Comparison between PHACCS + GAAS/BLAST and ENVirT estimates of viral richness and average genome length on viral metagenomes derived from different environments

Source	Sample name	ENVirT			PHACCS		
		*L*	*M*	Evenness	*L* ^*§*^	*M*	Evenness
French Lakes	Lake Bourget	62279	42999	0.84862	13089 ^⋆^	33311	0.92228
[6]	Lake Pavin	81110	792	0.82202	12274 ^⋆^	2628	0.89747
Feitsui	V1	24112	587	0.84216	44297 ^⋆^	3059	0.72402
Reservoir	V2	16613	1288	0.88611	43926 ^⋆^	513	0.93042
[17]	V3	31019	617	0.93707	95269 ^⋆^	174	0.94079
	V4	16535	1092	0.89225	62395 ^⋆^	399	0.91161
	V5	15177	1121	0.89919	41377 ^⋆^	419	0.93946
	V6	46677	1929	0.79735	125321 ^⋆^	221	0.90320
Fermented	Shrimp	27337	4931	0.92204	39839 ^*†*^	4606	0.90349
food	Kimchi	53837	1395	0.88842	48220 ^*†*^	1415	0.89653
[32]	Sauerkraut	277163	719	0.80599	36494 ^*†*^	2692	0.86619
Perennial ponds	Ilij	75242	1703	0.88137	71477 ^⋆^	1687	0.88550
of the	Molomhar	394921	223	0.87082	60959 ^⋆^	1318	0.89228
Mauritanian Sahara	Hamdoun	176346	515	0.66600	60479 ^⋆^	217	0.88719
[5]	El Berbera	81118	6199	0.69961	76501 ^⋆^	5696	0.71009
Human	X-1	175863	559	0.83496	50000 ^*‡*^	815	0.92174
gut	H1-1	497223	609	0.62918	50000 ^*‡*^	397	0.92259
[28]	H1-2	387877	212	0.73163	50000 ^*‡*^	353	0.92904
	H1-7	282786	151	0.78132	50000 ^*‡*^	315	0.92531
	H1-8	570706	121	0.68525	50000 ^*‡*^	239	0.94400

*M* = estimated richness, *L* = estimated average genome length (bp). *§* = Average genome length used in the original publication. ⋆= An estimate based on GAAS software ([33]). *†*= An estimate based on a BLAST search. *‡*= Assumed value

### Simulations

To objectively evaluate the performance of ENVirT in comparison to PHACCS and Catchall, we artificially constructed viromes under two simulation scenarios. These simulation scenarios were designed to mimic the variability that has been observed in real-world data sets.

When estimating viral richness using virome data, the evenness of the underlying populations plays a critical role in arriving at robust estimates. Evenness in the context of a viral population is given by the following equations:

9 $$ \text{evenness} = \frac{-{\sum\nolimits}_{i=1}^{M} f_{i}.ln(f_{i})}{ln(M)}  $$

where *f*_*i*_ is the relative abundance of the *i*^*th*^ genotype (*i*∈1,2,3,…*M*). Evenness measures whether the population is skewed toward a particular set of dominant viral types (i.e. when evenness approaches 0) or whether all viral types are equally abundant (i.e. when evenness approaches 1); the range of possible evenness values for any given population is (0,1].

#### Simulation scenario 1:

We simulated mixtures of viral populations that had varying degrees of viral richness and evenness, subject to the constraint that each viral type has the same average genome length. For all simulated mixtures, the contig spectra were generated based on a total read count of 10,000, a read length of 100bp and a minimum overlap of 35bp. These parameters were chosen in accordance with the default parameters used by Circonspect.

#### Simulation scenario 2:

As an extension to Simulation Scenario 1, we simulated mixtures of viral populations that not only had varying degrees of richness and evenness, but also variable genome lengths. This simulation scenario is more in-line with the expected characteristics of real-world samples. Here we assumed that genome lengths are normally distributed with $\mathcal {N}\left (L,(L.v)^{2}\right)$ where *L* denotes the average genome length and *v* denotes the coefficient of variation of the considered genome length distribution.

## Results

### Simulating viral mixtures

Quantitatively comparing the accuracy of viral richness estimates requires data that represents ground truth. We generated such data sets by simulating viromes based on a known number of viral genotypes, average genome length and relative abundance distribution. This simulation study follows the same methodology as previous studies of PHACCS and CatchAll [3, 8, 11]. We provided PHACCS with the true average genome length (*L*), whereas ENVirT was required to estimate *L* based only on the simulated data itself. We note that this benchmark study is highly advantageous to PHACCS in that PHACCS is given critical information that ENVirT will be required to estimate.

#### Simulation scenario 1 (fixed average genome length):

We divided these simulations into two groups. First, we limited the range of simulations to a set of 16 conservative benchmark data sets that represent low to moderately complex viral mixtures. As an extension to these results, we then show how the algorithms perform on a wider simulation range of 77 benchmark data sets that were designed to identify the parameter limits at which reliable estimates are attainable for each method.

We observed that ENVirT produced an average estimation error of 0.91%, whereas PHACCS produced an average estimation error of 585.87% (Table 3). We also see that ENVirT is better able to optimize the model parameters, and select the most appropriate relative abundance model in all 16 simulations. In several cases, PHACCS and CatchAll were not able to produce any reasonable estimates. In accordance with previous reports, CatchAll produced significant overestimates when a discounted parametric model was not selected by its internal model selection procedure [15, 16].

**Table 3:** Performance comparison between ENVirT, PHACCS and CatchAll on simulated contig spectra

Input parameters (expected result)						ENVirT				PHACCS				CatchAll
*L*_0_	*M*_0_	*T*_0_	*d*_0_	Evenness	*f*_*max*_	*M*	*T*	*d*	*S*_*min*_	*M*	*T*	*d*	*S*_*min*_	*M*
12500	300	exp	0.030	0.790	2.956%	300	exp	0.030	0.00x10 ^0^	4096	exp	0.030	1.37x10 ^-3^	2829.6 ^*p*^
12500	1000	log	0.900	0.995	0.661%	1000	log	0.900	0.00x10 ^0^	1000	log	0.900	0.00x10 ^0^	92628.3 ^*c*^
12500	5000	lgn	2.500	0.655	11.849%	5000	lgn	2.500	0.00x10 ^0^	23563	pl	1.313	1.01x10 ^4^	3246.1 ^*p*^
12500	10000	pl	0.700	0.913	1.997%	10000	pl	0.700	0.00x10 ^0^	10000	pl	0.700	0.00x10 ^0^	696.3 ^*p*^
50000	300	exp	0.030	0.790	2.956%	300	exp	0.030	0.00x10 ^0^	10000	exp	0.030	4.31x10 ^-4^	15712.6 ^*p*^
50000	1000	log	0.900	0.995	0.661%	1000	log	0.900	0.00x10 ^0^	1000	log	0.900	0.00x10 ^0^	n/a
50000	5000	lgn	2.500	0.655	11.849%	5000	lgn	2.500	0.00x10 ^0^	4996	lgn	2.500	1.78x10 ^-3^	799.8 ^*p*^
50000	10000	pl	0.700	0.913	1.997%	10000	pl	0.700	0.00x10 ^0^	10000	pl	0.700	0.00x10 ^0^	413688.9 ^*c*^
125000	300	exp	0.030	0.790	2.956%	300	exp	0.030	0.00x10 ^0^	10000	exp	0.060	1.87x10 ^-4^	70340.9 ^*c*^
125000	1000	log	0.900	0.995	0.661%	1000	log	0.900	0.00x10 ^0^	1000	log	0.900	0.00x10 ^0^	n/a
125000	5000	lgn	2.500	0.655	11.849%	5000	lgn	2.500	0.00x10 ^0^	5000	lgn	2.500	0.00x10 ^0^	2303.2 ^*p*^
125000	10000	pl	0.700	0.913	1.997%	10000	pl	0.700	0.00x10 ^0^	10000	pl	0.700	0.00x10 ^0^	n/a
300000	300	exp	0.030	0.790	2.956%	300	exp	0.030	0.00x10 ^0^	4096	exp	0.030	7.92x10 ^-5^	160243.9 ^*c*^
300000	1000	log	0.900	0.995	0.661%	1000	log	0.900	0.00x10 ^0^	1000	log	0.900	0.00x10 ^0^	n/a
300000	5000	lgn	2.500	0.655	11.849%	5000	lgn	2.500	0.00x10 ^0^	5000	lgn	2.500	0.00x10 ^0^	146552.7 ^*c*^
300000	10000	pl	0.700	0.913	1.997%	8547	pl	0.689	3.00x10 ^-3^	10000	pl	0.700	0.00x10 ^0^	n/a

Contig spectra were generated with parameters: *R*=10000, *r*= 100bp and *o*= 35bp. Both ENVirT and PHACCS were provided with the true average genome length (*L*_0_) value. pl = power-law distribution, exp = exponential distribution, log = logarithmic distribution and lgn = lognormal distribution. *S*_*min*_ = the value of the cost function corresponding to the estimated values of *M*,*T* and *d* for each method. For each spectrum, the CatchAll estimate having the minimum error compared to *M*_0_ is reported. ^*p*^ = best discounted parametric model produced by CatchAll. ^*c*^ = Chao1 non-parametric estimate. n/a denotes samples for which CatchAll failed to produce an output

**Fig. 1:**
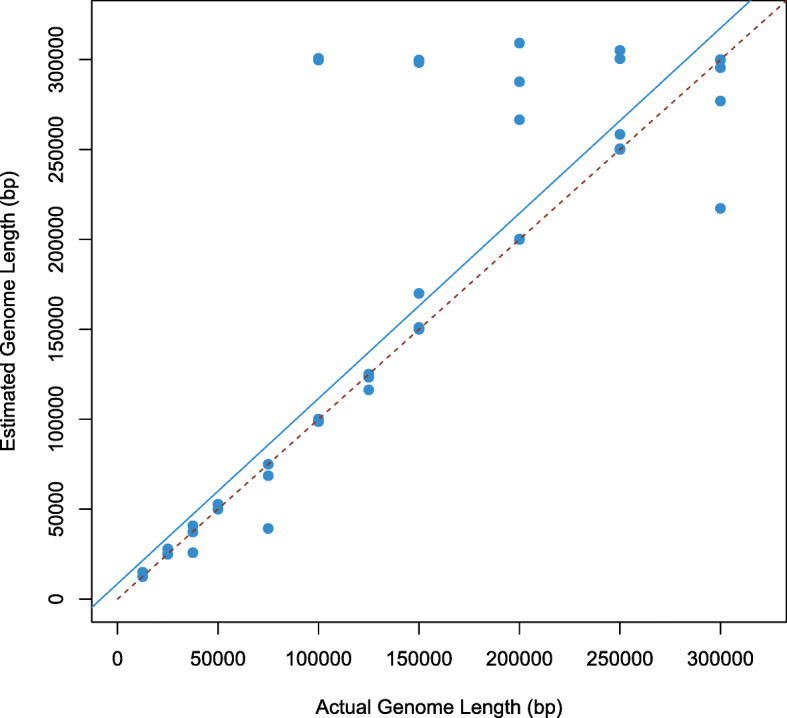
Estimated average genome length versus true average genome length for ENVirT. This analysis uses only the information contained in the contig spectra for a given virome. As such, it is shown that ENVirT does not require underlying sequence data or other databases to estimate an average genome length. Here, ENVirT is able to estimate the true average genome length with an average error of 9.13*%*

The results for the extended 77 data sets indicate a similar trend as the initial set of 16 simulations, with ENVirT outperforming both PHACCS and CatchAll (Figure S4 of Additional file 2). We found that at certain extremes, neither PHACCS nor CatchAll was able to produce an estimate of viral richness. Interestingly, these instances tend to underestimate richness (up to − 83.48*%*) as the number of viral types increases. In contrast, PHACCS tends to largely overestimate richness when the true value of richness is in the range of 300-10,000. ENVirT is stable at both extremes (low and high values of richness), and has a maximum estimation error of only − 16.58*%* at a richness value of 45,000. We also note that when estimating an average genome length using only information contained in the aggregate statistics of contig spectra, ENVirT performs with an average error of 9.13% (Fig. 1). ENVirT’s estimation accuracy of *L* tends to fall at *L* values greater than 100 kbp compared to lower *L* values. Other methods such as GAAS use sequence similarity to compare the DNA sequence data of a virome to known viral sequences in databases. Since ENVirT and these database-dependent methods use fundamentally different approaches and entirely different types of data to estimate an average genome length, we do not directly compare these methods here.

#### Simulation scenario 2 (variable genome lengths):

In general, it is reasonable to assume that constituent viruses in a virome do not have the same genome length. To evaluate how our method performs in comparison to PHACCS in this regard, we generated an additional 140 contig spectra representing populations with predefined degrees of genome length variation. The resulting spectra represent populations with viral genome lengths distributed according to: *N*(*L*,(*Lv*)^2^), where *L* denotes the average genome length and *v* is the coefficient of variation. For comparison between PHACCS and ENVirT, we considered only a power-law distribution as the model for relative viral abundance to ensure that viral types of lower abundance are captured by each respective model. The results indicate that for both PHACCS and ENVirT there is an estimation error that increases exponentially with the increase in genome length variation *v*. Notably, we observed that ENVirT is more performant relative to PHACCS at larger values of variation. ENVirT is up to 55.62% more stable than PHACCS in the presence of genome length variation at lower viral richness (*M*=300) and 9.80% more stable at higher viral richness (*M*=10,000).

**Fig. 2:**
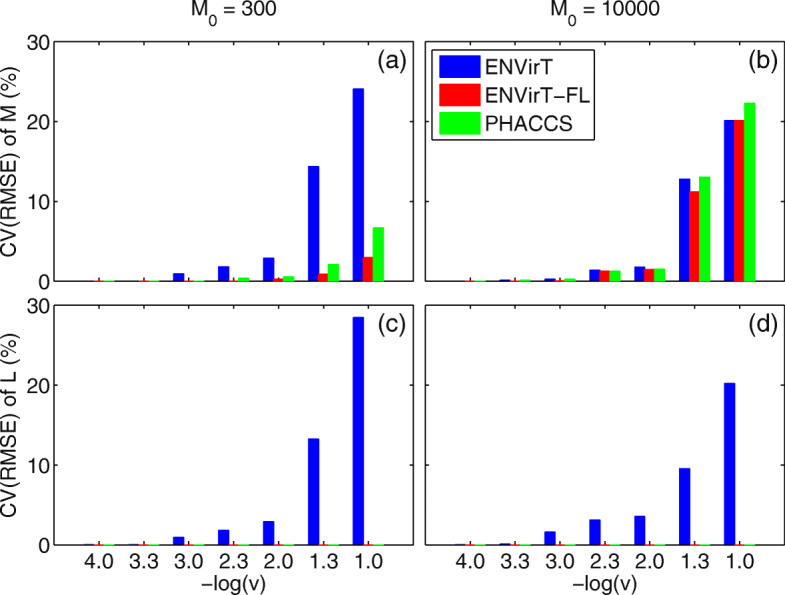
**a** CV(RMSE) of estimated *M* when *M*_0_=300, **b** CV(RMSE) of estimated *M* when *M*_0_=10000, **c** CV(RMSE) of estimated *L* when *M*_0_=300 and **d** CV(RMSE) of estimated *L* when *M*_0_=10000: of spectra in Simulation Scenario 2 categorized under different values of *v* (*v*∈{0.0001,0.0005,0.001,0.005,0.01,0.05,0.1}). CV(RMSE) = Coefficient of Variation of the Root Mean Squared Error, *M*_0_ = simulated true richness, *M* = estimated richness and *L* = estimated average genome length. All 140 spectra used here were derived from populations simulated using a genome length distribution of $\mathcal {N}(L_{0},(L_{0}.v)^{2})$ and *L*_0_=50 kbp. ENVirT-FL = ENVirT algorithm given a fixed value for *L*. Only the power-law distribution was considered in all three methods. Values summarized in the figure are given in Table S1 of Additional file 1)

With respect to ENVirT’s estimation of *L* in the presence of a wide variation of genome length (i.e. when −*log*(*v*)<2), we note that the error of the estimates produced increases. Conversely, if the expected variation between genome lengths is sufficiently small (i.e. −*log*(*v*)>2), ENVirT is observed to produce more robust estimates (Fig. 2).

Normal distributions with different variances were selected as the simplest and appropriate forms to model real world scenarios. Two alternative approaches that may be taken to model genome length distributions are the use of existing data and the use of in vitro method, flow cytometry. However, mentioned alternative approaches pose limitations. The existing data on genome lengths of viruses are limited and their use to derive the viral genome length distributions may be inaccurate. Flow cytometry may be used to visualize the distribution of particle sizes of a sample of viruses [27] and may be used to infer the viral genome length distribution in vitro. However, conducting multiple experiments with flow cytometry is quite expensive, constrained by the limited availability of machines and no experiment has been conducted for this purpose as yet to the best of our knowledge.

### Running Time Comparison

The running time of ENVirT is directly proportional to the number of genotypes in the population for given values of *L*_*LB*_, *L*_*UB*_, *d*_*LB*_, *d*_*UB*_ and *N*_*L*_. To compare the computational efficiency of the methods, we analyzed simulated contig spectra of popuations with low-high richness having parameters: *M* = [5000, 45000, 100000], *L* = 50 kbp, power law distribution and *d* = 0.7. The experiment specific parameters were: *R* = 10000, *r* = 100 bp and *o* = 35 bp. Input parameters to ENVirT were given as follows: *M*_*LB*_= 1, *M*_*UB*_= 120000, *L*_*LB*_= 15 kbp, *L*_*UB*_= 75 kbp, *d*_*LB*_= 0.01, *d*_*UB*_= 5 and *N*_*L*_= 5. Under these parameters, ENVirT consumed 35.57, 62.17 and 85.77 mins of wall clock time to anlayze contig spectra of populations having richness of 5000, 45000 and 100000 respectively, over the four types of relative abundance distributions from 15 kbp to 75 kbp. The running time of PHACCS is directly proportional to the number of genotypes in the population. To analyze the same three contig spectra, PHACCS consumed 44.52, 150.8 and 167.6s of wall clock time respectively, over the four types of relative abundance distributions when it is provided with the correct average genome lengths (L) of the population. Therefore, to analyze contig spectra of populations with richness of 5000, 45000 and 100000 over the average genome lengths (L) from 15 kbp to 75 kbp to determine the best estimate for L, PHACCS would take 742, 2513.3 and 2793.3 h respectively. Hence, ENVirT would take only 0.04 - 0.08% of the time taken by an approach based on PHACCS to analyze a contig spectrum over a range of L to determine the best estimate for the average genome length of a contig spectrum without making unfair or biased predictions. These measurements were taken on a desktop computer running Windows 7 (64-bit) operating system on an Intel Core i7-4790 CPU @3.60 GHz with 16 GB RAM. The contig spectra were trimmed to a length of 50 before analyzing using PHACCS, because PHACCS could not find the optimal results with the original contig spectra.

### Analysis of 20 experimental viromes

To validate ENVirT and its applicability to experimental viromes, we repeated the analysis of 20 samples using ENVirT and compared the results with those obtained using PHACCS, as well as previously conducted in vitro analysis of each respective sample. As required by PHACCS, we provided the algorithm with either GAAS or BLAST-based estimates of genome length to estimate viral richness. We then compared this combination of PHACCS + GAAS/BLAST with ENVirT. We note that we have excluded CatchAll from all subsequent comparisons, since it has been shown to perform poorly on all our simulations. For data sets where contig spectra needs to be re-calculated, we used the contig spectrum generation software Circonspect (version 0.2.6, https://sourceforge.net/projects/circonspect/) with parameter settings as described in Online Methods. Figure S5 of Additional file 2 shows that the number of iterations used by Circonspect to generate the contig spectra is sufficient.

A summary of the parameter estimates produced by ENVirT and PHACCS is presented in Table 2 and depicted in Figure S6 of Additional file 2. As a measure of performance, we use the value of *S*_*min*_ (the minimum value of the cost function described in Eq. 7), which represents the residual error of a generated model. The ideal value of *S*_*min*_ is 0, which corresponds to a perfect model of the observed virome data. We found that in all cases, ENVirT clearly outperforms PHACCS + GAAS/BLAST in terms of this performance metric (Figure S7 of Additional file 2).

#### Lake Bouget and Lake Pavin:

Average genome length values of 13,089bp and 12,274bp as reported by GAAS, were used in the analysis for Lake Bourget and Lake Pavin respectively. Using these genome length estimates, PHACCS estimated viral richness to be 33,331 and 2628, respectively. In contrast, ENVirT produced estimates of viral richness, with a much smaller model error, of 42,999 and 792 respectively. We used Circonspect to compute the contig spectra over 1000 iterations, where each iteration considered 10,000 reads (Figure S5 of Additional file 2). Moreover, we see a 5-fold difference in the genome length estimates produced by ENVirT (62.2 kbp and 81.1 kbp) relative to GAAS, which in turn, explains the difference in diversity estimates produced by PHACCS.

#### Fetsui Reservoir, North Taiwan:

The six reservoir samples, V1-V6 were sampled before and after the occurrence of typhoons over a 2-year period. As such, it is expected that the diversity estimates will vary in accordance with these seasonal disturbances, as observed in the richness estimates of ENVirT and PHACCS. We observed that GAAS produced much larger estimates of average genome length based on hits to similar viruses. ENVirT produced a much tighter range of richness estimates (587-1929) over all samples, whereas PHACCS estimated a much broader range of richness (174-3059).

#### Fermented food:

A BLAST search was used to identify closely related viral types for each of the Shrimp, Kimchi and Sauerkraut samples to estimate the respective average genome lengths. This produced estimates of 39.8 kbp, 48.2 kbp and 36.4 kbp based on similar viruses. The relative differences between richness estimates between PHACCS and ENVirT were 6.59% and 1.43% for the Shrimp and Kimchi samples, respectively. However, ENVirT estimated the average genome length of the Sauerkraut sample to be 277.1 kbp, in contrast to the BLAST-based estimate of 36.5 kbp. This is reflected in ENVirT’s lower richness estimates. This critical difference is explained in the subsequent discussion.

#### Mauritanian Sahara:

Four perennial pond samples were analyzed. The Ilij, Hamdoun and El Berbera samples were in close agreement with previously reported richness estimates. However, of particular interest is the Molomhar sample, which had a predicted richness of 223 by ENVirT and 1318 by PHACCS. We also note that ENVirT estimated a much larger average genome length of 394.92 kbp in comparison to the re-calculated GAAS estimate of 60.96 kbp. The much lower model error produced by ENVirT suggests that it was better able to estimate a more representative viral richness.

#### Human gut:

An assumed average genome length value of 50 kbp was used for all human gut samples as per the recommendations of the original study [28]. The overall richness of all 5 samples was relatively lower than the other samples that were analyzed. However, there was a close agreement between the order of magnitude of the richness estimates produced by ENVirT and PHACCS. However, the observed genome length estimates produced by ENVirT clearly indicate the presence of much larger genomes (175.6 - 570.7 kbp).

## Discussion

We have clearly demonstrated in the benchmark analysis using simulated data that ENVirT can estimate viral richness with a higher accuracy and computational efficiency than PHACCS, despite providing additional information that advantaged the latter. To the best of our knowledge, we have also demonstrated for the first time that additional databases are not required to infer the average genome length for an experimental sample. This is in contrast to the widely used PHACCS algorithm which relies on other methods, such as GAAS and BLAST, as well as other databases to analyze experimental data.

The formulation of ENVirT can be considered as an extension to the original model used by PHACCS but is still fundamentally different. ENVirT estimates thus are uncorrelated with the estimates generated by PHACCS, hence there is no systematic bias in our extended model compared to the original model. When benchmarked against simulated data, the proposed extensions allow ENVirT to accurately estimate an average genome length with an average error of 9.13%, while at the same time being 9.80-55.62% more accurate than PHACCS in the presence of genome length variation and up to 66.90% more accurate than PHACCS and CatchAll at varying levels of viral richness.

However, we did observe that there is a significant reduction in estimation accuracy for all methods when the evenness of a viral population approaches one (i.e. all viruses are equally abundant; *d*=0; refer Eqs. 1 to 4). This translates to an optimization landscape for Eq. 7 that has multiple global minima, meaning that there are multiple equally valid solutions. To some degree, the proposed niching strategy of ENVirT is able to find a global minima that is close to the desired solution, but Additional file 1 shows that when evenness is equal to one, a single solution to the minimization of *S* (Eq. 7) does not exist.

Our analysis of 20 experimental viromes revealed unique insights into each of the underlying viral populations. In all cases, we found that the results produced by ENVirT were more consistent with the findings of each respective study than the results produced by PHACCS + GAAS/BLAST. For instance, a common observation among these analyses is that larger viral genomes were not considered when estimating richness using PHACCS + GAAS/BLAST. Our analyses show that this behaviour can skew richness estimates to the point where very different conclusions can be drawn from that data.

This is most notable in the Sauerkraut sample which contains viruses that have much larger genomes, including T4-like viruses, SOP1-like viruses and Mimiviruses, as identified using MEGAN [29]. This sample also contains many unclassified viruses, which we expect to be larger viruses. While ENVirT is able to correctly account for these viruses, PHACCS + GAAS/BLAST is unable to do so. Instead, it predicts a viral mixture containing much smaller viral genomes. This is then reflected in very different richness estimates.

Similar results were obtained for the Human Gut samples when analyzed using ENVirT. In this instance, larger estimates of average genome length could be indicative of host DNA contamination or gene transfer agents that had likely affected the samples [28, 30]. Bacterial species, *Mycoplasma* with larger genome lengths (> 0.5 - 1 Mb) may not be removed using a 0.2 *μ*m filter. In fact, unknown viruses had previously been excluded from downstream computational analyses, which could include much larger viruses [28] that could pass through the filter. Again these findings could be not observed using PHACCS + GAAS/BLAST. This also suggests the importance of a methodology to learn the variation of genome lengths of a virome which has not been addressed by ENVirT or PHACCS. Although the exact reason for the observation of average viral genome lengths larger than 500 kbp is unknown, the results suggest that large viruses might be more common in human gut vial assemblages than our current understanding.

Mesotrophic lakes, such as Lake Bourget, are expected to be much more nutrient rich than oligotrophic lakes like Lake Pavin and hence contain higher microbial and viral prevalence [6]. This hypothesis was confirmed by both ENVirT and PHACCS. However, ENVirT was better able to optimize a population model than PHACCS + GAAS/BLAST, suggesting that previous estimates did not capture the full extent of the relative diversity of both lakes.

The Feitsui Reservoir samples were collected based on the hypothesis that viral diversity increases after a typhoon [17]. The original study confirmed that terrestrial viruses infiltrate these marine communities, contributing to larger average genome lengths. This phenomena was corroborated by ENVirT but could not be fully explained using PHACCS + GAAS/BLAST. For example, a high proportion of *Mimiviridae* and *Phycodnaviridae* were detected in sample V6. These correspond to relatively large viral taxa, which should skew the average genome length to much larger values. This is true for ENVirT but not for PHACCS + GAAS/BLAST. Moreover, smaller viral taxa (*Circoviruses*, *Nanoviruses* or *Microviruses*) were detected in samples V2, V4 and V5, which is again in agreement with ENVirT estimates. As a result, we see that ENVirT is better able to optimize the corresponding population models at much lower residual error than PHACCS + GAAS/BLAST.

Previous results based on Transmission Electron Microscopy for the Molomhar samples had identified relatively large *Mimivirus*-like particles (3~00nm viral particles) [5]. This agrees with the average genome length estimates produced by ENVirT. Additional in vitro analysis of the Sahara and Namib samples revealed higher molecular weight DNA (270 - 350 kbp) than other samples, again confirming ENVirT’s estimates of viruses with distinctively larger genome lengths in both samples. These larger viral genomes were not represented in the results produced by PHACCS + GAAS/BLAST.

The technique of Multiple Displacement Amplification (MDA) used in sample preparation prior to sequencing may have introduced biases towards certain viral types which could affect the estimated values of species richness obtained by ENVirT and PHACCS [31]. In this study, metaviromic data sets from the French lakes, Feitsui reservoirs, Sahara desert and human gut were all amplified using MDA. In their current versions both PHACCS and ENVirT cannot rule out this bias. Owing to the improvement of sequencing such that the most recent platforms require much less DNA concentration, MDA would not be necessary in the preparation of viral DNA, and the bias will no longer be problematic in species richness estimation.

We note that ENVirT is not capable of inferring the variations in genome lengths of a given virome. There are several possible extensions to ENVirT that could alleviate this limitation and enhance its performance on experimental data. For example, a phage community could have several different discrete and dictating genome lengths such as 5 kbp, 50 kbp, 100 kbp and 200 kbp. Reformulating ENVirT’s objective function to account for variations in genome length rather than assuming a point estimate could improve richness estimation accuracy over ENVirT in such scenarios. Section Simulation Scenario 2 shows how ENVirT and PHACCS deviate from expected estimates when such variability is present in the virome. Additional heuristics could also be implemented to reduce the computational cost of the algorithm. For instance, GA could be applied to a number of sub-spaces considerably less than 4*N*_*L*_ based on the features of the local optima found at the search space boundaries of *S*(*M*,*L*,*T*,*d*). The suggested extensions shall also include a strategy to improve the estimation accuracy of *L* when *L*>100 *kbp*. It is also worth exploring how ENVirT can be improved to analyze contig spectra generated from recently introduced assemblers such as de-bruijin graph assemblers, to increase the applicability of ENVirT.

## Conclusions

Estimating the parameters that describe a viral community underpins our ability to deeply understand viral ecology. In this regard, ENVirT is shown to be faster and more accurate than the most performant algorithm that has previously been developed on simulated benchmark datasets. Moreover, ENVirT does not rely on reference databases to estimate viral richness or an average genome length for novel experimental data. We have evaluated the performance of ENVirT on simulated data, highlighting its improvement and utility over existing methods. We have also demonstrated its validity in analyzing 20 experimental samples from a wide range of environments, revealing unique insights that were previously not observed. ENVirT is thus set to be an essential tool for studying viral ecology.

## Additional files

Additional file 1 Supplementary Methods. Detailed description of ENVirT methodology. (PDF 822 kb)

Additional file 2 Supplementary Results. Supplementary figures and tables. (PDF 234 kb)
