# Supplementary material for: ENVirT: inference of ecological characteristics of viruses from metagenomic data
Source: BMC Bioinformatics. 2019 Feb 4;19(Suppl 13):377. doi: 10.1186/s12859-018-2398-5 (PMC7394321; doi:10.1186/s12859-018-2398-5)
Supplement: Supplementary file 1 — Supplementary Methods. Detailed description of ENVirT methodology. (PDF 822 kb) [file 12859_2018_2398_MOESM1_ESM.pdf]

Supplementary File 1 - Supplementary Methods

Assume the following parameters for a given viral metagenome.

M = Number of genotypes (richness)
L = Average genome length of each genotype (bp)
f_i = Relative abundance of the ith genotype ($i \in 1, ..., M$)
R = Number of reads
r = Read length (bp)
o = Minimum overlap distance considered in assembling reads (bp)
$(C_1, C_2, C_3, ..., C_R)$ = Observed contig spectrum, where C_q ($q \in 1, 2, 3, ..., R$) is the observed number of contigs each having exactly q reads.
$O_q = q.C_q$ = Number of reads out of the total R that contributed to observed contigs that have exactly q reads ($q \in 1, 2, 3, ..., R$).

An important assumption made in this formulation is that the f_is follow one of the four theoretical distributions: power-law, exponential, logarithmic or lognormal.

If f_is have a power-law distribution;

$$f_i = \frac{i^{-d}}{\sum_{j=1}^{M} j^{-d}} \text{ where } d \geq 0 \tag{1}$$

If f_is have an exponential distribution;

$$f_i = \frac{exp(-i.d)}{\sum_{j=1}^{M} exp(-j.d)} \text{ where } d \geq 0 \tag{2}$$

If f_is have a logarithmic distribution;

$$f_i = \frac{(log(i+1))^{-d}}{\sum_{j=1}^{M} (log(j+1))^{-d}} \text{ where } d \geq 0 \tag{3}$$

If f_is have a lognormal distribution;

$$f_i = \frac{exp(m_i.d)}{\sum_{j=1}^{M} exp(m_j.d)} \text{ where } d \geq 0 \tag{4}$$

$m_i = \frac{M}{\sqrt{2\pi}}.\left(exp\left(\frac{-t_i^2}{2}\right) - exp\left(\frac{-t_{i+1}^2}{2}\right)\right)$

$t_1 = -\infty$, $t_{M+1} = +\infty$, $t_{i+1} = \sqrt{2}.erf^{-1}\left(\frac{2}{M} + erf\left(\frac{t_i}{\sqrt{2}}\right)\right)$ where erf denotes the error function and erf^{-1} denotes the inverse error function.

All four functional forms of f_i (i.e. equations 1, 2, 3 and 4) depends on M and a distribution specific parameter d. Let us denote the function giving the relative abundance of the ith genotype as $F_i(M, T, d)$ where T denotes the distribution function.

If the expected number of reads contributing to contigs having exactly q number of reads is E_q ($q \in \{1, 2, 3, ..., R\}$);

$$E_q = \sum_{i=1}^{M} F_i(M, T, d).R.q.p_i^{(q-1)}.(1 - p_i)^2 \tag{5}$$

where,

$$p_i = 1 - exp\Big(- (r-o).F_i(M,T,d).\frac{R}{L}\Big) \tag{6}$$

Accordingly, the expected contig spectrum of a metagenome having population parameters M, L, T, d and, sequenced and assembled with parameters R, r, o is; $\Big(\frac{E_1}{1}, \frac{E_2}{2}, \frac{E_3}{3}, ..., \frac{E_R}{R}\Big)$.

Given the values of R, r, o and $(O_1, O_2, O_3, ..., O_R)$, our aim is to find the values of M, L, T and d such that the difference between $(O_1, O_2, O_3, ..., O_R)$ and $(E_1, E_2, E_3, ..., E_R)$ is minimum.

We use the variance weighted squared difference between $(O_1, O_2, O_3, ..., O_R)$ and $(E_1, E_2, E_3, ..., E_R)$ denoted by $S(M, L, T, d)$ as the similarity measure between the observed and expected contig spectra.

$$S(M,L,T,d) = \sum_{q=1}^{R} \frac{(O_q - E_q)^2}{V_q^2} \tag{7}$$

where,

$$V_q^2 = \sum_{i=1}^{M} F_i(M,T,d).R.q.p_i^{(q-1)}.(1-p_i)^2.\Big(1 - q.p_i^{(q-1)}.(1-p_i)^2\Big) \tag{8}$$

$S(M, L, T, d)$ has multiple local minima and one global minimum with highly similar characteristics for given values of R, r, o and $(C_1, C_2, C_3, ...)$. Consequently, our goal now is to find the values of M, L, T and d when $S(M, L, T, d)$ is at its global minimum.

In order to understand the effect of the presence of multiple local minima, let us consider a population where $d = 0$. For any case of T, $F_i(M, T, 0) = \frac{1}{M}$. In other words $d = 0$ corresponds to a population where all M number of genotypes are equally abundant (this is a highly unlikely scenario in a real population). Let us simplify above equations for $d = 0$.

Equation 6 simplifies to

$$p_i = p = 1 - exp\Big(- (r-o).\frac{R}{L.M}\Big) \tag{9}$$

Therefore, p is independent of i and depends only on the product term $L.M$.

Accordingly, equation 5 simplifies to

$$E_q = R.q.p^{(q-1)}.(1-p)^2 \tag{10}$$

Simplified E_q depends only on p which is a function of $L.M$.

This result implies that, for a given sample, $(E_1, E_2, E_3, ..., E_R)$ will be identical for different L and M value pairs satisfying the equation $L.M = constant$. If L_0 and M_0 are the true average genome length and the true number of genotypes respectively of the given sample, then $S(M_0, L_0, T, 0) = 0$. Furthermore, $S(M, L, T, 0) = 0$ for all M and L value pairs such that $M.L = M_0.L_0$ (i.e. $S(M, \frac{M_0.L_0}{M}, T, 0) = 0$). Hence, $S(M, L, T, 0)$ have identical multiple minima along the curve $M.L = M_0.L_0$ making it impossible to find a single pair of M and L values that minimize $S(M, L, T, 0)$. Figures S1 and S2 shows an example of this scenario where we observe identical local minima when $d = 0$.

When $d > 0$, there still exists multiple local minima in $S(M, L, T, d)$ but the values differ from $S(M_0, L_0, T, d) = 0$. Also, the relationship $M.L = M_0.L_0$ does not necessarily hold at local minima when $d > 0$. Figure S2 shows an example of how the cost function $S(M, L, T, d)$ varies over the region $1000 \leq M \leq 50000$, $5000 \leq L \leq 100000$ and $d \in \{0.6, 0.7, 0.8\}$ for a simulated contig spectrum with parameters $M_0 = 10000, L_0 = 50000bp, T_0 = power - law, d_0 = 0.7, R = 10000, r = 100bp$ and $o = 40bp$ (subscript $_0$ indicates the true value used to simulate the population). We observe that, when $d = 0.7$ (Figure 3(c)) there exist multiple local minima and a unique global minimum having the value 0. When $d \neq 0.7$ (Figures 3(a) and 3(e)), there still exist multiple local minima and a unique global minimum having values greater than 0. Hence, empirically we observe that for populations with $d > 0$, there exist a unique global minimum with $S(M, L, T, 0) = 0$ at M_0, L_0, T_0 and d_0. Therefore, a unique global minimum is expected to be found when $d > 0$ even in the presence of multiple local minima. However, finding the unique global minimum cannot be guaranteed using a heuristic algorithm without utilizing appropriate niching strategies.

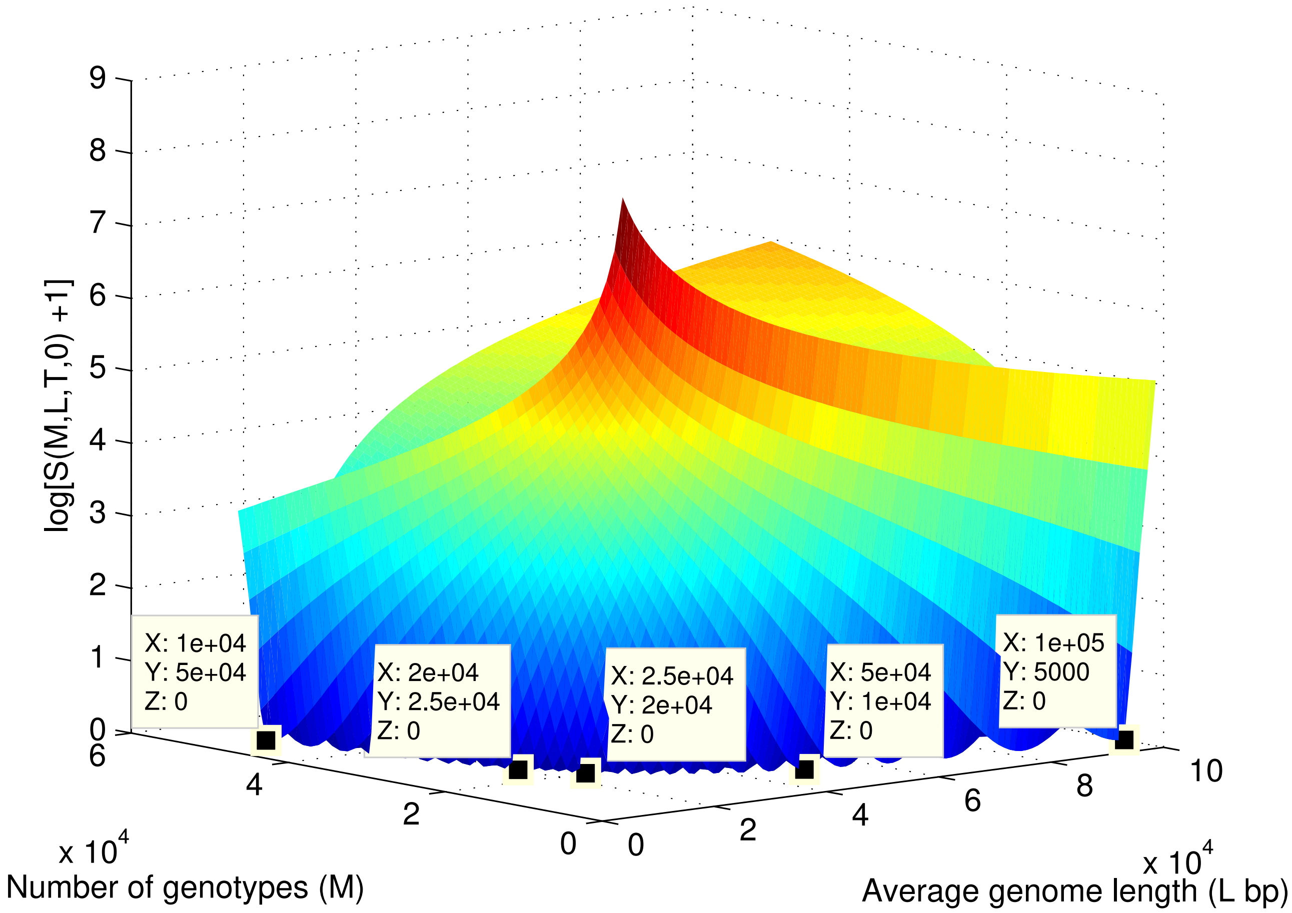

Figure S1: Surface plot of $log(S(M, L, T, 0) + 1)$ over the region $100 \leq M \leq 50000$ and $5000 \leq L \leq 160000$. The observed contig spectrum used here is a simulated spectrum with parameters $M_0 = 10000, L_0 = 50000bp, d_0 = 0, R = 10000, r = 100bp, o = 40bp$ (subscript $_0$ indicates the true value used to simulate the population). $log(S(M, L, T, 0) + 1)$ is plotted instead of $S(M, L, T, 0)$ for the ease of demonstration. The global minimum points are indicated with a cursor points.

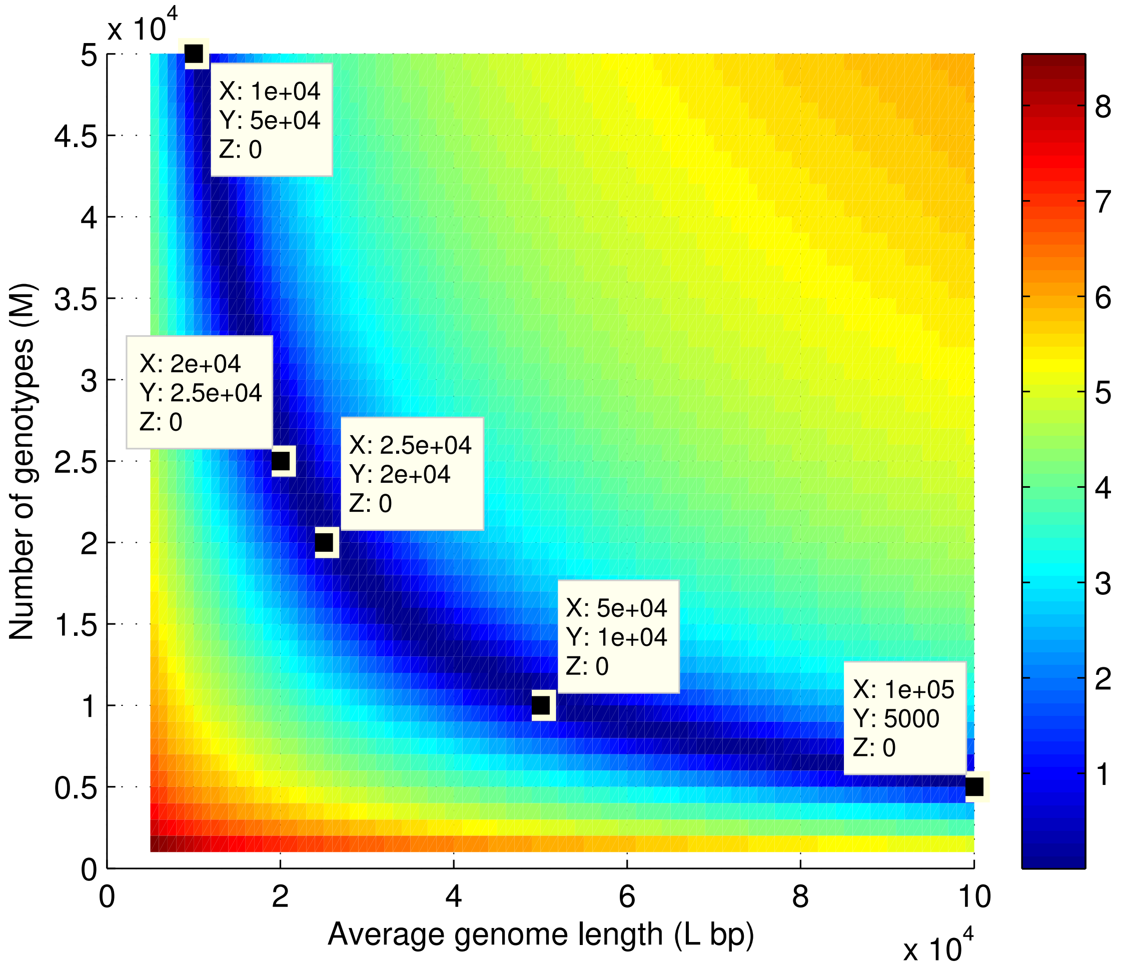

Figure S2: Heat map of $log(S(M, L, T, 0) + 1)$ over the region $100 \leq M \leq 50000$ and $5000 \leq L \leq 160000$. The observed contig spectrum used here is a simulated spectrum with parameters $M_0 = 10000, L_0 = 50000bp, d_0 = 0, R = 10000, r = 100bp, o = 40bp$ (subscript $_0$ indicates the true value used to simulate the population). $log(S(M, L, T, 0) + 1)$ is plotted instead of $S(M, L, T, 0)$ for the ease of demonstration. The global minimum points are indicated with a cursor points.

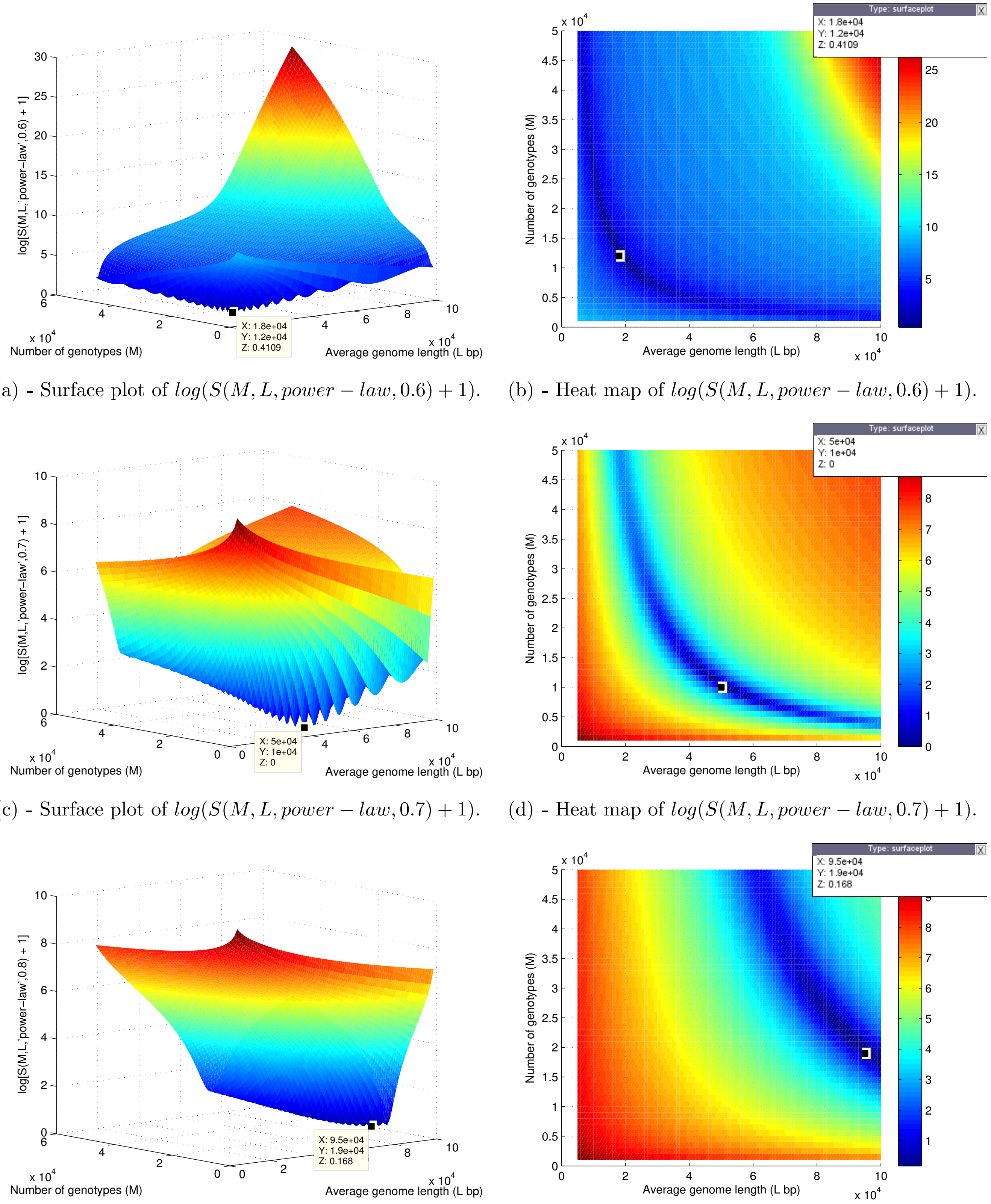

(a) - Surface plot of $log(S(M, L, power-law, 0.6)+1)$. (b) - Heat map of $log(S(M, L, power-law, 0.6)+1)$.

(c) - Surface plot of $log(S(M, L, power-law, 0.7)+1)$. (d) - Heat map of $log(S(M, L, power-law, 0.7)+1)$.

(e) - Surface plot of $log(S(M, L, power-law, 0.8)+1)$. (f) - Heat map of $log(S(M, L, power-law, 0.8)+1)$.

Figure S3: Surface plots and heat maps of $log(S(M, L, power-law, d)+1)$ over the region $1000 \leq M \leq 50000$, $5000 \leq L \leq 100000$ and $d \in \{0.6, 0.7, 0.8\}$. The observed contig spectrum used here is a simulated spectrum with parameters $M_0 = 10000, L_0 = 50000bp, T_0 = power-law, d_0 = 0.7, R = 10000, r = 100bp, o = 40bp$ (subscript $_0$ indicates the true value used to simulate the population). $log(S(M, L, power-law, d)+1)$ is plotted instead of $S(M, L, power-law, d)$ for the ease of demonstration. The global minimum point of each plot is indicated with a cursor point.
