# Supplementary material for: ENVirT: inference of ecological characteristics of viruses from metagenomic data
Source: BMC Bioinformatics. 2019 Feb 4;19(Suppl 13):377. doi: 10.1186/s12859-018-2398-5 (PMC7394321; doi:10.1186/s12859-018-2398-5)
Supplement: Supplementary file 2 — Supplementary Results. Supplementary figures and tables. (PDF 234 kb) [file 12859_2018_2398_MOESM2_ESM.pdf]

Supplementary File 2 - Supplementary Results

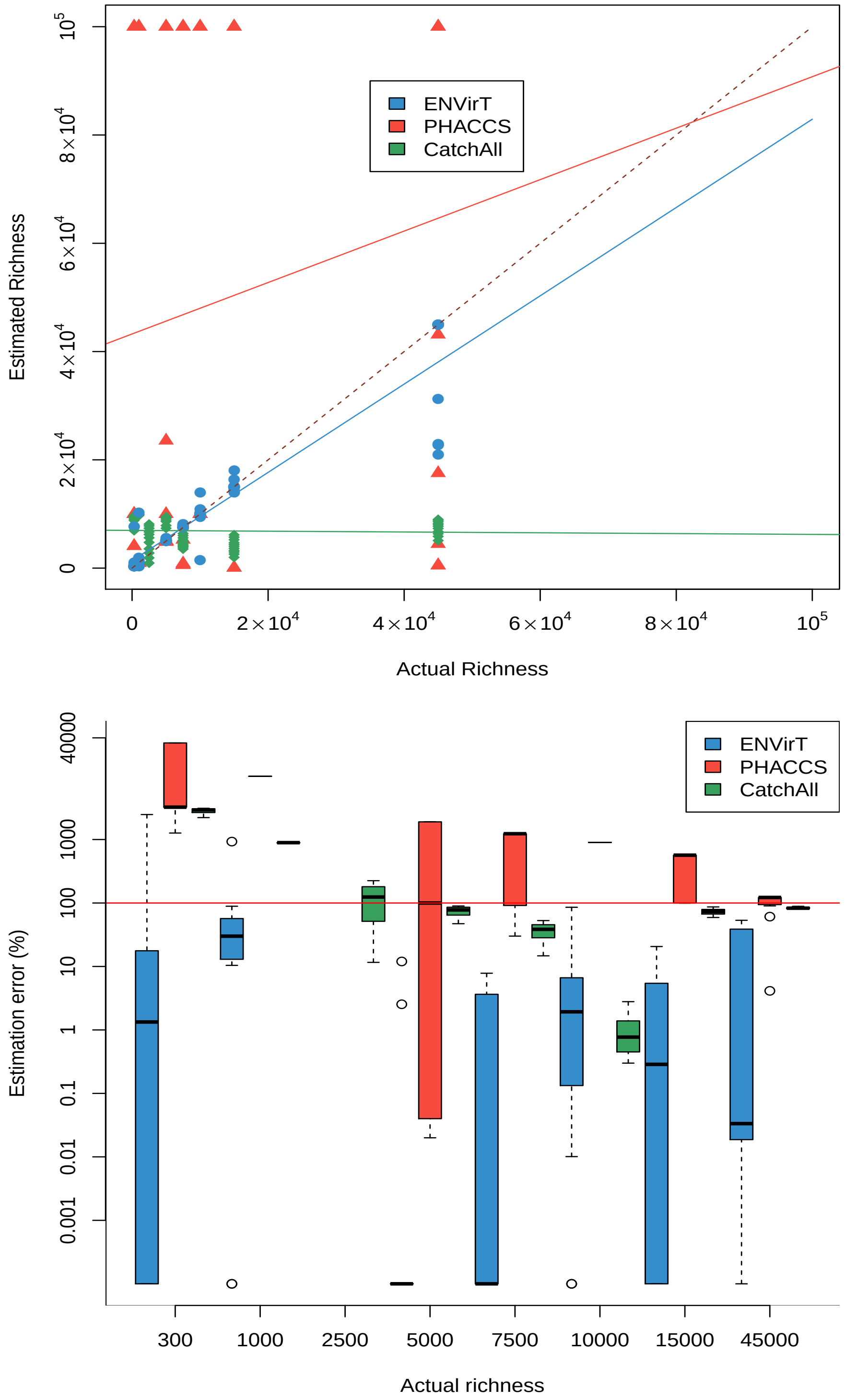

Figure S4: (top) Estimated richness (M) vs. True richness (M_0) under different average genome lengths (L); (bottom) Estimation error vs true richness. Note that estimation error values are plotted in a log2 scale for comparison between the larger errors produced by PHACCS in relation to ENVirT. On average we see that ENVirT performs up to 585% more accurately than PHACCS.

Table S1: CV(RMSE) values for L and M estimates for M = 300 and M = 10000 for different v values. The results are obtained by running the same dataset on ENVirT, ENVirT-FL and PHACCS. N/A - Not Applicable

		CV(RMSE) of L estimate			CV(RMSE) of M estimate		
Scenario	-log(v)	ENVirT	ENVirT-FL	PHACCS	ENVirT	ENVirT-FL	PHACCS
M = 300	4	0.00002	N/A	N/A	0.00000	0.00000	0.00000
	3.3	0.00007	N/A	N/A	0.00000	0.00000	0.00000
	3	0.00935	N/A	N/A	0.00913	0.00000	0.00000
	2.3	0.01828	N/A	N/A	0.01814	0.00000	0.00365
	2	0.02894	N/A	N/A	0.02896	0.00258	0.00548
	1.3	0.13252	N/A	N/A	0.14370	0.00882	0.02098
	1	0.28470	N/A	N/A	0.24046	0.02961	0.06672
M=10000	4	0.00018	N/A	N/A	0.00024	0.00000	0.00023
	3.3	0.00105	N/A	N/A	0.00130	0.00021	0.00112
	3	0.01595	N/A	N/A	0.00252	0.00042	0.00266
	2.3	0.03104	N/A	N/A	0.01363	0.01251	0.01246
	2	0.03581	N/A	N/A	0.01766	0.01468	0.01524
	1.3	0.09553	N/A	N/A	0.12784	0.11198	0.13040
	1	0.20204	N/A	N/A	0.20084	0.20102	0.22287

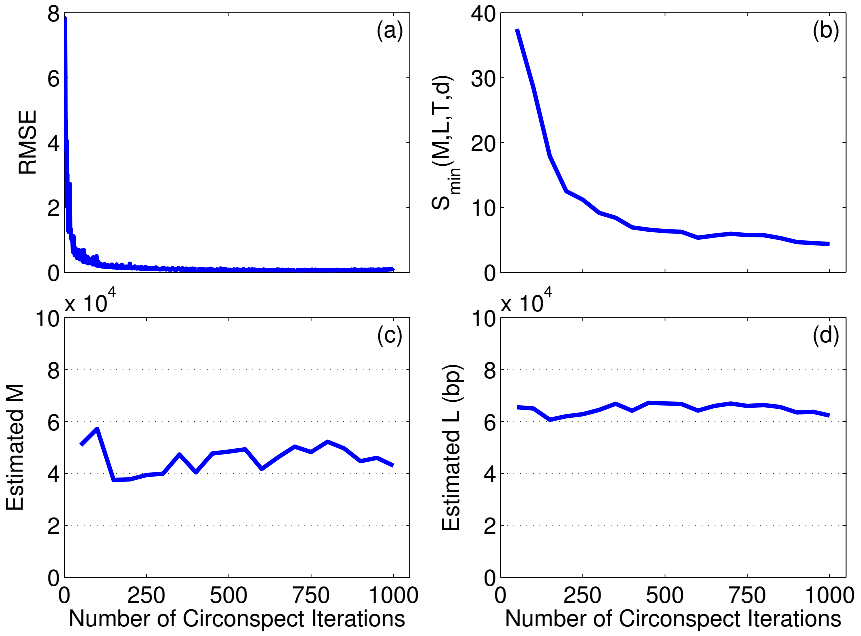

Figure S5: (a) Root Mean Squared Error (RMSE) between the average contig spectra of two consecutive iterations, (b) S(M,L,T,d) corresponding to the estimates, (c) Estimated M and (d) Estimated L (bp): produced by ENVirT under the contig spectrum of Lake Bourget averaged over different numbers of Circonspect iterations.

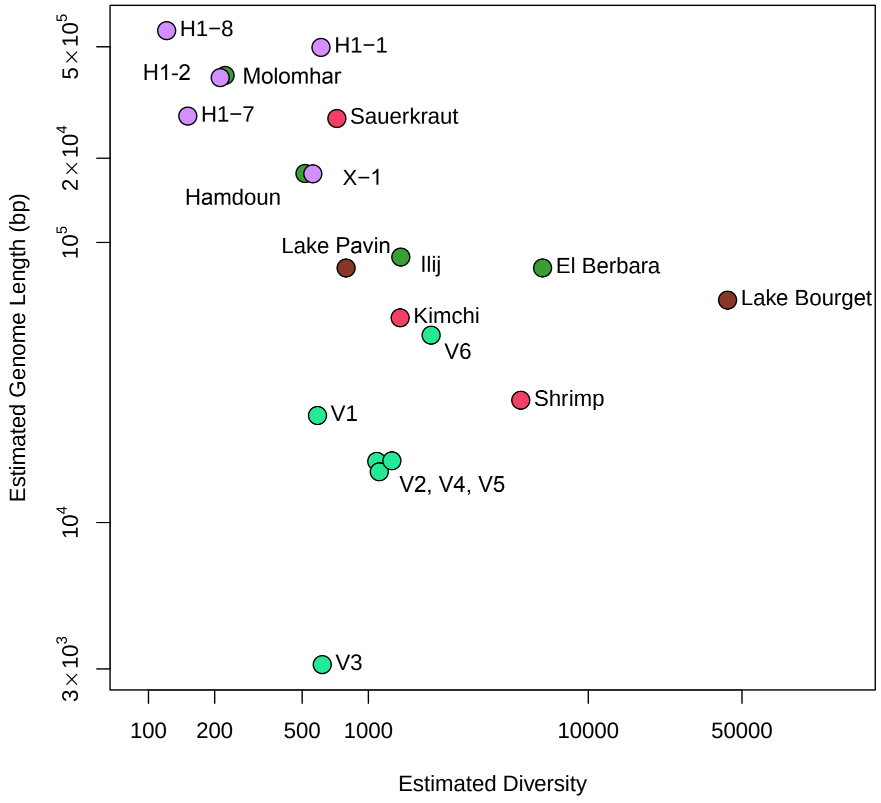

Figure S6: Estimated richness and average genome length as generated by ENVirT for 20 experimental metaviromes.

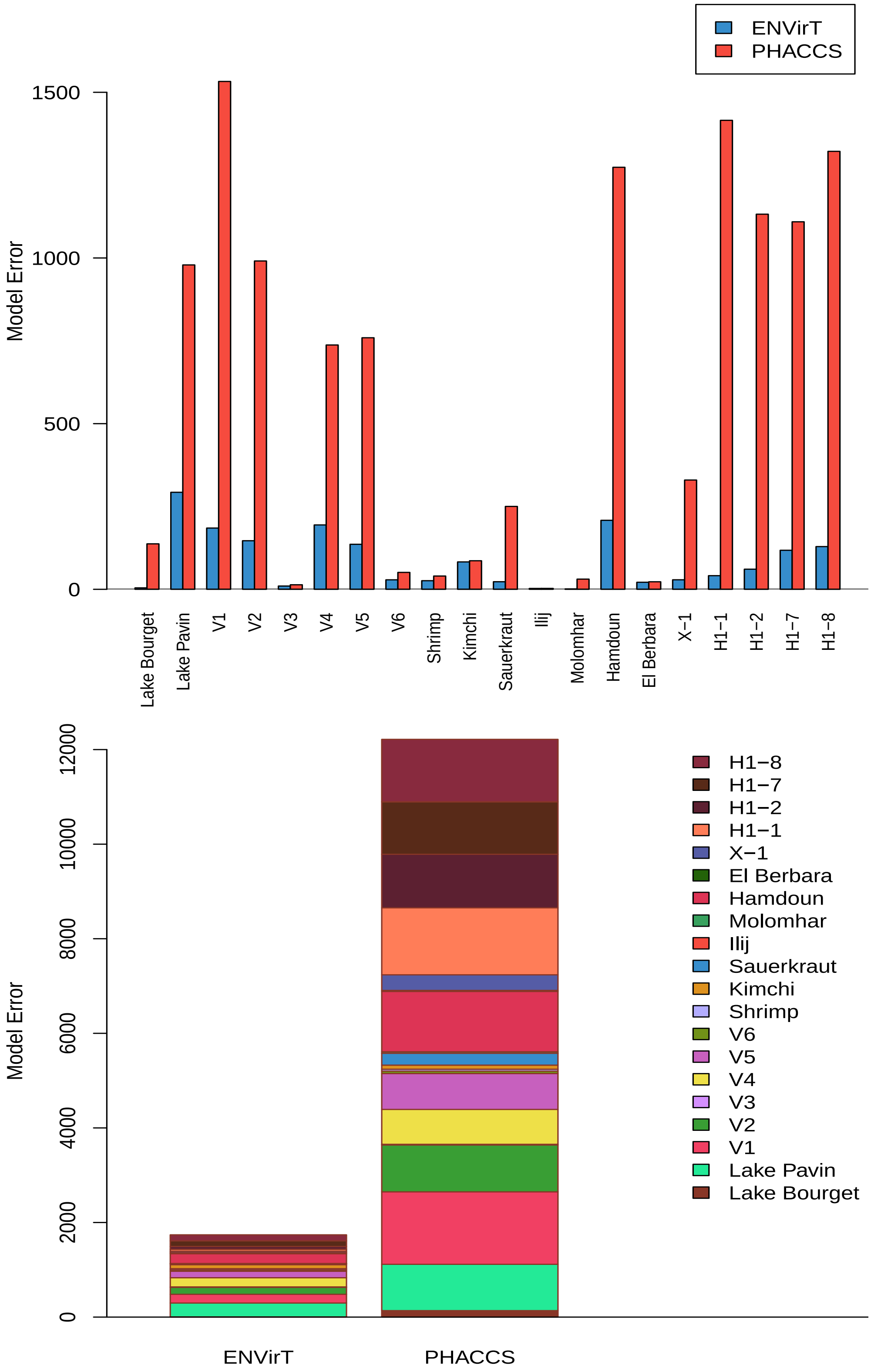

Figure S7: (top) Comparison of the residual model error as given by S_{min} between ENVirT and PHACCS+GAAS/BLAST; (bottom) The cumulative model error S_{min} for all 20 experimental metaviromes analyzed by both ENVirT and PHACCS+GAAS/BLAST.
